# Quantifying the degree of white spot lesions on enamel caused by different commercial beverages using the Canary Caries Detection System: An in vitro study

**DOI:** 10.34172/joddd.2022.005

**Published:** 2022-05-29

**Authors:** John Michaelis, Qingzhao Yu, Tom Lallier, Xiaoming Xu, Richard W Ballard, Paul Armbruster

**Affiliations:** ^1^Orthodontist, Private practitioner, New Orleans, USA; ^2^Department of Biostatistics, LSUHSC School of Public Health, New Orleans, USA; ^3^Department of Cell Biology and Anatomy, LSUHSC School of Dentistry, New Orleans, USA; ^4^Department of Prosthodontics, LSUHSC School of Dentistry, New Orleans, USA; ^5^Department of Orthodontics, LSUHSC School of Dentistry, New Orleans, USA

**Keywords:** Canary system, Demineralization, Energy beverages, Sports beverages

## Abstract

**Background.** The use of sports and energy drinks has drastically increased in the adolescent population. This population often is in orthodontic treatment, and the use of such drinks with poor oral hygiene promotes the development of white spot lesions (WSLs). Quantifying the degree of the lesion has been limited in the past. The hypothesis was that the Canary Caries Detection System could be used to quantify the degree of WSLs caused by different commercial beverages.

**Methods.** A total of 105 extracted human premolars were divided into seven groups (n=15). Each group was tested in one of six beverages or a phosphate-buffered solution (control). The teeth were exposed to its beverage three times a day for 15 minutes for 28 days. Canary numbers and ambient light and fluorescent photographs were collected at baseline (T_0_ ) and on days 14 (T_1_ ) and 28 (T_2_ ).

**Results.** The paired *t* test and one-way ANOVA found that T_0_ to T_1_ measurements were statistically significant (*P*<0.0015) and that T_0_ to T_2_ measurements were statistically significant (*P*<0.0001). Visually, the ambient light photographs and fluorescent photographs from T_0_ to T_1_ and T_1_ to T_2_ correlated with the increase in Canary numbers.

**Conclusion.** This in vitro study revealed a statistically significant increase in the T_0_ to T_1_ Canary numbers and a statistically significant increase from T_0_ to T_2_ Canary numbers for all the test beverages. Changes in Canary numbers indicated significant changes in mineral density (i.e., demineralization) and development of WSLs on enamel after exposure to sports and energy beverages.

## Introduction

 The majority of people who seek orthodontic treatment have the same goal. They want straight, beautiful teeth. The patient may not obtain that goal if they do not have adequate oral hygiene. Poor oral hygiene and plaque buildup on the teeth can lead to white spot lesion (WSL) formation on the enamel surface of teeth.^1^ WSLs are the early demineralization of enamel that may or may not progress to caries. Fixed appliances and the bonding materials increase the potential for the retention of biofilm, which could lead to WSLs.^2^ Clinically visible WSLs have been reported to occur in approximately 23% and 28% of orthodontic patients treated in the university and private practice.^3^

 The combination of bad oral hygiene and plaque buildup can be exacerbated by continually consuming beverages throughout the day. Sports drinks and energy drinks such as Bodyarmor^®^Fruit Punch, Powerade^®^ Fruit Punch, Gatorade^®^ Lemon-lime, Monster^®^Energy, Bang^®^ Rainbow Unicorn, and Red Bull^®^ Regular have become choices over water.^4^ Some individuals consume these beverages multiple times a week.^5^ The 2015 Youth Risk Behavior Survey found that 31.8% of adolescents consumed 1 to 3 sports drinks in the past week, 11.9% consumed 4–6 sports drinks in the past week, and 3.2% consumed ≥4 sports drinks daily.^6^ The use of carbonated drinks ≥4 times a week has been reported to significantly increase the risk of developing WSLs compared with less frequent usages of such beverages.^7^ These beverages decrease the pH in the oral cavity, allowing dental plaque to flourish. The increase in dental plaque in the presence of an acidic environment and cariogenic sugars results in an imbalance of the demineralization and remineralization balance that exists in a healthy mouth. This imbalance is first visualized as WSLs and can progress to frank dental caries.^8^

 There are multiple caries detection devices to evaluate the extent of enamel demineralization. Two that are commercially available are DIAGNOdent (Kavo Dental, Brea, CA, USA and the Canary System (Quantum Dental Technologies Inc., Toronto, Ontario, Canada). The Canary System has exhibited a much higher correlation with caries lesion depth and higher sensitivity for caries detection than DIAGNOdent.^9^ The Canary System uses an intraoral camera and low-power laser to detect caries. The pulses of laser light generate photothermal and luminescence responses. According to the Canary System website,^10^ by using a laser pulse at a frequency of 2 Hz, the laser light can penetrate below the tooth surface and permit the detection of a carious lesion as small as 50 μm and as deep as 5 mm from the tooth surface. A “Canary number” is then produced from the gathered information. The number can range from 0 to 100, with the healthy range being 0-20, the decay range being 21-70, and the advanced decay range being 71-100. This research aimed to assess the effect of six popular beverages (three sports drinks and three energy drinks) on enamel using natural light and fluorescence photography and to use the Canary System to quantify the enamel demineralization from these beverages. This study aimed to quantify the degree of WSLs on enamel caused by different commercial beverages using the Canary Caries Detection System.

## Methods

 This study utilized 105 extracted non-carious, deidentified, human premolar teeth. Exclusion criteria for the sample teeth included restorations on the facial surface, visible caries, and enamel fractures under ×2.5 magnification.

 Each tooth was numbered 1 through 105 on the root surface. A 3×3-mm sticker was placed at the center of the facial surface of each tooth, and then the facial surface was covered with a layer of clear nail polish. Once thoroughly dried, the sticker was removed, leaving a 3×3-mm test area of unprotected enamel. The test area then received a Canary System measurement. The test area of each tooth was measured three times and then averaged together to form the mean Canary number for that tooth. The test area of each tooth was also initially photographed with an Apple iPhone 7 (Apple, Cupertino, CA, USA) under natural ambient lighting (T_0_). The facial surfaces were also photographed using a Nikon inverted TE2000-S fluorescence microscope (Nikon, Chiyoda, Japan) with a BV-2A fluorescent filter combination (420 ± 20 nm/470 nm) (T_0_).

 The teeth were stored in a 1% thymol solution for 72 hours. After being rinsed in distilled water for 10 seconds, the 105 premolars were randomly divided into seven groups (n = 15). The group numbers and beverage test solutions were as follows.

- Group 1: Bodyarmor^®^ Fruit Punch
- Group 2: Powerade^®^ Fruit Punch
- Group 3: Gatorade^®^ Lemon-lime
- Group 4: Monster^®^ Energy
- Group 5: Bang^®^ Rainbow Unicorn
- Group 6: Red Bull^®^ Regular
- Group 7: Phosphate-buffered solution (control)

The phosphate-buffered solution (PBS) was prepared using 0.2 g of KCl, 0.2 g of KH_2_PO_2_, 8 g of NaCl, 1.15 g of NaH_2_PO_4_, 10 g of thymol, and 1000 mL of distilled and deionized water.

 Each test group was exposed to its test liquid for 15-minute sessions, three times a day, with 5–7-hour intervals between each session at 20°C. This would mimic drinking three beverages per day. After each exposure to the test liquid, the teeth were rinsed with distilled water before being stored in phosphate-buffered solution at 20°C. New bottles of each beverage were used at each test session to ensure adequate carbonation (if present) and no dilution. The control group remained in the PBS at 20°C. The PBS used for the control and the PBS used as a storage for the test groups were changed daily. The mean pH of each test liquid was determined using an electronic pH meter and measuring the pH of three individual bottles of each beverage. After 14 days (T_1_) and 28 days (T_2_) of testing, each tooth was again subjected to ambient light photographs of the facial surface test area, fluorescent photographs of the facial surface test area, and new Canary number readings. Again, the test area of each tooth was recorded three times and averaged together to form the Canary number.

## Results

 A normality test showed that all six beverages and PBS were normally distributed. Table 1 presents the means and standard deviations obtained from the pH testing of each beverage. The pH values of groups 3, 4, and 6 were slightly lower than Reddy’s findings.^11^ Group 2 had a higher pH compared to Reddy’s findings.^11^ The mean pH of the groups ranged from 4.96 to 2.79. A paired t-test comparing the T_0_ to T_1_ measurements, as shown in Table 1, concluded that there was a statistically significant difference in the mean from T_0_ Canary numbers to T_1_ Canary numbers for groups 1 (*P* = 0.0002), 2 (*P* = 0.0015), 3 (*P* < 0.0001), 4 (*P* < 0.0001), 5 (*P* = 0.0002), 6 (*P* < 0.001), and 7 (*P* < 0.05). All initial Canary numbers changed from the healthy range to the decay range according to the Canary Scale.

**Table 1:** Beverages, pH, average Canary values, value changes, and ranks

**Drink Type**	**Group Number**	**Beverage**	**pH**	**Average Canary Value**			**Change in Canary Value**			**pH Rank**	**T0-T1 Rank**	**T1-T2 Rank**	**T0-T2 Rank**
				**T0**	**T1**	**T2**	**T0-T1 Change**	**T1-T2 Change**	**T0-T2 Change**				
**Sports Drinks**	1	Bodyarmor^®^ Fruit Punch	3.82	17.29	26.60	43.87	9.31	17.27	26.58	6	3	1	1
	2	Powerade^®^ Fruit Punch	2.9	17.51	22.24	38.29	4.73	16.05	20.78	3	6	2	3
	3	Gatorade^®^ Lemon-lime	2.79	17.53	27.38	35.09	9.85	7.71	12.56	1	2	5	5
**Energy Drinks**	4	Monster^®^ Energy	3.47	17.62	31.22	40.60	13.60	9.38	22.98	5	1	4	2
	5	Bang^®^ Unicorn Rainbow	2.83	18.18	24.00	30.40	5.82	6.40	12.22	2	5	6	6
	6	Red Bull^®^ Regular	3.36	17.22	25.64	37.07	8.42	11.43	19.58	4	4	3	4
**Control**	7	Phosphate Buffer Solution	4.96	20.00	23.38	23.82	3.38	0.44	3.82	7	7	7	7

 Another paired t-test comparing the T_0_ measurements with T_2_ measurements, as shown in Table 1, concluded that there were statistically significant differences between groups 1 (*P* < 0.0001), 2 (*P* < 0.0001), 3 (*P* < 0.0001), 4 (*P* < 0.0001), 5 (*P* < 0.0001), 6 (*P* < 0.0001), and 7 (*P* < 0.001). Again, there was a statistically significant change in Canary numbers when T_0_ and T_2_ measurements were compared.

 A one-way ANOVA test compared the intra-group change of T_0_ measurements to T_1_ measurements and concluded that there were statistically significant differences between all groups, including the control group. At T_1_, group 4 had the highest mean Canary number change, and group 2 had the least among the experimental groups (Table 2). Another one-way ANOVA compared the intra-group changes from T_0_ to T_2_, indicating a statistically significant difference between all the groups, including the control group. Group 1 had the greatest mean Canary number change from T_0_ to T_2_, and group 5 had the least among the experimental groups (Table 3).

**Table 2:** ANOVA to compare the change from T_0 _to T_1_, showing a statistically significant difference between each individual beverage

**Group**	**Analysis variable: T**_0_ ** to T**_1_					
	**No. of observations**	**Mean**	**Standard deviation**	**Minimum**	**Maximum**	***P*** ** value**
Group 1	15	9.311	7.401	-6.667	20.333	< 0.001
Group 2	15	4.733	4.649	-3.000	12.000	< 0.001
Group 3	15	9.844	4.537	-0.333	16.333	< 0.001
Group 4	15	13.600	5.470	6.667	26.333	< 0.001
Group 5	15	5.822	4.565	-0.333	17.333	< 0.001
Group 6	15	8.422	3.043	4.667	15.667	< 0.001
Group 7 (Control)	15	3.377	1.122	-0.333	10.667	< 0.001

**Table 3:** ANOVA to compare the change from T_0_ to T_2_, showing a statistically significant difference between each individual beverage

**Group**	**Analysis variable: T**_0_ ** to T**_2_					
	**No. of observations**	**Mean**	**Standard deviation**	**Minimum**	**Maximum**	***P*** ** value**
Group 1	15	26.578	5.242	17.333	34.667	< 0.001
Group 2	15	20.778	5.144	9.667	29.667	< 0.001
Group 3	15	17.556	3.031	11.000	22.667	< 0.001
Group 4	15	22.978	4.056	18.000	31.667	< 0.001
Group 5	15	12.222	7.523	0.00	27.000	< 0.001
Group 6	15	19.844	5.735	7.667	29.667	< 0.001
Group 7 (Control)	15	3.822	2.357	-0.333	7.000	< 0.001

 The ANOVA test showed a significant inter-group difference at T_1_ (*P* < 0.0001). The Tukey test results are shown in Table 4. Compared with the control group, groups 1, 3, and 4 had significantly greater changes from T_0_ to T_1_. The ANOVA test also showed a significant inter-group difference at T_2_ (*P* < 0.0001). The Tukey results are shown in Table 5. Compared with the control group, all the groups had significantly higher changes from T_0_ to T_2_.

**Table 4:** Comparison of inter-group differences at T_1_

**Tukey grouping**			**Mean**	**N**	**Group**
	A		13.6	15	Group 4
B	A		9.8444	15	Group 3
B	A		9.3111	15	Group 1
B	A	C	8.4222	15	Group 6
B		C	5.8222	15	Group 5
B		C	4.7333	15	Group 2
		C	3.3778	15	Group 7 (control)

N = Number, means with the same letter are not significantly different from each other.

**Table 5:** Comparison of inter-group differences at T2

**Tukey grouping**		**Mean**	**N**	**Group**
	A	26.5778	15	Group 1
B	A	22.9778	15	Group 4
B		20.7778	15	Group 2
B		19.8444	15	Group 6
B	C	17.5556	15	Group 3
	C	12.2222	15	Group 5
	D	3.8222	15	Group 7 (Control)

N = Number, Means with the same letter are not significantly different from each other.

## Discussion

 Energy and sports drinks have turned into a multi-billion-dollars-a-year industry.^12^ Bodyarmor^®^ Fruit Punch, Monster^®^ Energy, Gatorade^®^ Lemon-lime, Powerade^®^ Fruit Punch, Bang^®^ Rainbow Unicorn, and Red Bull^®^ Regular were selected to represent each category. Red Bull^®^ Regular, Monster^®^ Energy, and Bang^®^ Rainbow Unicorn were selected to represent energy drinks in this study because they held a 46.2% United States market share in 2020.^13^ Powerade^®^ Fruit Punch and Gatorade^®^ Lemon-lime dominate the sports drink market with over $7 billion in sale for 2020.^12^ Bodyarmor^®^ Fruit Punch is relatively new to the market and is a new competitor for Powerade^®^ Fruit Punch and Gatorade^®^ Lemon-lime. Due to Bodyarmor^®^ Fruit Punch being new to the market, it was chosen to represent the sports drinks along with Powerade^®^ Fruit Punch and Gatorade^®^ Lemon-lime.

 Consumption ofsports drinks and energy drinks has increased in children and adolescents with multiple drinks throughout the day.^14^ This finding influenced the design of our study to include exposure to the test liquids three times a day, and that consumption would take 15 minutes. All the test liquids were found to have a pH below 5.5, the highest pH at which enamel demineralization can be seen.^15^ The average of the initial Canary numbers for all tooth samples ranged from 17.22 to 20.0 (Table 1). According to the Canary Scale, this puts all the tooth samples in the healthy/sound tooth structure range.

**Figure 1:**
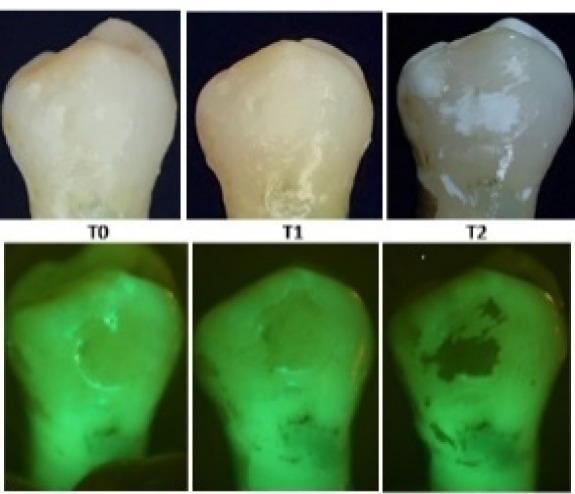
The white light and fluorescent light images at T_0_, T_1_, and T_2_ for the sample subjected to Bodyarmor® Fruit Punch. This tooth’s Canary numbers were 18.67 (T_0_), 26.33 (T_1_), and 49.33 (T_2_).

**Figure 2:**
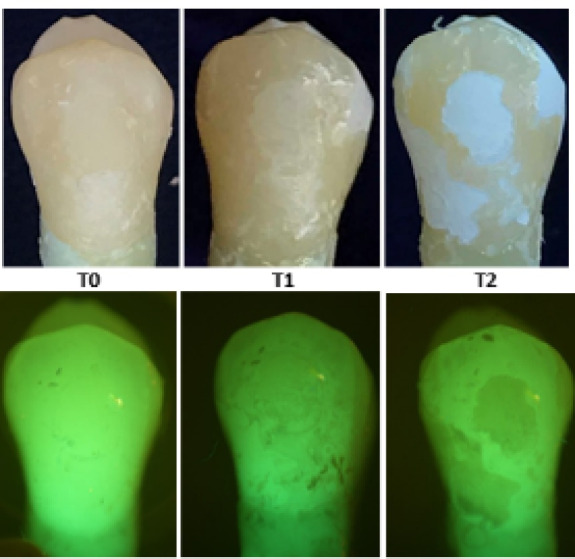
The white light and fluorescent light images at T_0_, T_1_, and T_2_ for the sample subjected to Bang® Unicorn Rainbow. This tooth’s Canary numbers were 19.67 (T_0_), 24.33 (T_1_), and 35.33 (T_2_).

**Figure 3:**
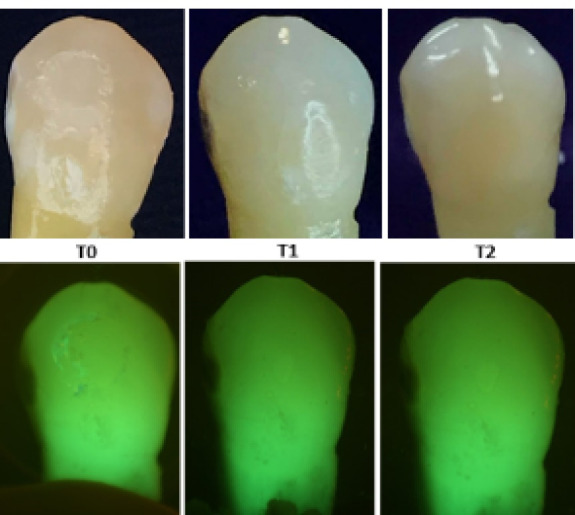
The white light and fluorescent light images at T_0_, T_1_, and T_2_ for the sample subjected to PBS. This tooth’s Canary numbers were 20.00 (T_0_), 21.00 (T_1_), and 22.00 (T_2_).

 At T_1_, the results exhibited a statistically significant increase in Canary numbers across all the groups. Among the experimental groups, the mean Canary number increased the least in group 2 at 4.73 to the most in group 4 at 13.60 (Table 1). The ambient light and fluorescent light photographs of the buccal surface at the T_0_ to T_1_ showed the facial enamel surfaces starting to demineralize, supporting the increased Canary number measurements [Figures 1-3]. The averaged T_1_ Canary number of the beverages ranged from 22.24 (group 2) to 31.22 (group 4) (Table 2). This placed the Canary numbers in the decay portion of the Canary Scale but on the very low end of the decay range.

 At T_2_, the mean of Canary numbers ranged from 30.40 (group 5) to 42.87 (group 1) (Table 1). Among the experimental groups, the lowest mean Canary number change from T_0_ to T_2_ was group 5 at 12.22, and the highest mean Canary number change was group 1 at 26.57 (Table 1). The T_2_ Canary number averages fell in the decay portion of the Canary Scale. The T_2_ ambient light and fluorescent light photographs again showed the facial enamel surface’s increased demineralization (Figures 1-3).

 The lowest pH, group 2, did not directly correlate to the most demineralization and highest Canary number. Group 1 had the greatest demineralization and change in Canary number. Another study looked at different beverages’ pH and demineralization and found the same result of no correlation between the amount of enamel demineralization and pH.^16^ Perhaps something else in beverages could be contributing to the demineralization in conjunction with pH. Future studies would need to look at the ingredients of all the beverages and compare them to identify any trends that could correlate with the amount of demineralization.

 This study had some limitations. The WSLs that occur in the oral cavity result from acid-producing bacteria within the dental plaque. In this in vitro study, WSLs were artificially created by acidic beverages. However, this method has been used in previous demineralization studies as a valid technique for producing WSLs.^16^ Another limitation was the nail polish sealing the enamel surface around the test area. It is apparent in the photographs that some of the nail polish is missing on some samples at T_1_ and T_2_ time intervals. The nail polish was intended to seal the majority of the tooth surfaces to create a limited test area. Multiple layers should have been placed to minimize the loss of the nail polish to maintain a constant size of the test area. Fortunately, this effect is minimized since this study did not attempt to quantify a change in the surface area of the test site. Another limitation was an attempt to measure exactly the same spot with the Canary System. The test area should have stayed as a 3×3-mm circle. However, with the nail polish chipping, the test area expanded. To offset this, three Canary number readings were taken in the original test area and averaged.

## Conclusion

- The results of this in vitro study revealed a statistically significant increase in the T_0_ Canary numbers to T_1_ Canary numbers and a statistically significant increase from T_0_ Canary numbers to T_2_ Canary numbers for all the test beverages.
- The change in Canary number indicated significant changes in mineral density (i.e., demineralization) and development of WSLs on enamel after exposure to sports and energy beverages.
- There was no correlation between the lowest pH and the greatest mean change in Canary number.
- There was no significant difference between the six beverages in demineralization (i.e., all the six beverages had a negative impact on the enamel.

## Acknowledgments

 The authors are also grateful to Tommy Lam for his help as a student research assistant.

## Authors’ Contribution

 JM: Concept, design, literature search, experimental studies, data acquisition, and manuscript preparation. QY: Data analysis, statistical analysis, manuscript editing, and manuscript review. TL and XX: Design, data acquisition, manuscript editing, and manuscript review. RB and PA: Supervision, design, manuscript editing, and manuscript review.

## Funding

 None.

## Ethics Approval

 The study was approved by the Institutional Biosafety Committee, LSUHSC Health Sciences Center of New Orleans (IBC #19161).

## Competing Interests

 The authors declare that they have no known competing financial interests or personal relationships that could have influenced the work reported in this paper.
